# Uncertainty and variability in computational and mathematical models of cardiac physiology

**DOI:** 10.1113/JP271671

**Published:** 2016-06-09

**Authors:** Gary R. Mirams, Pras Pathmanathan, Richard A. Gray, Peter Challenor, Richard H. Clayton

**Affiliations:** ^1^ Computational Biology, Department of Computer ScienceUniversity of OxfordOxfordOX1 3QDUK; ^2^ US Food and Drug Administration10903 New Hampshire AvenueSilver SpringMD20993USA; ^3^ College of Engineering, Mathematics and Physical ScienceUniversity of ExeterExeterEX4 4QFUK; ^4^ Insigneo institute for in‐silico medicine and Department of Computer ScienceUniversity of SheffieldRegent CourtSheffieldS1 4DPUK

## Abstract

**Key points:**

- Mathematical and computational models of cardiac physiology have been an integral component of cardiac electrophysiology since its inception, and are collectively known as the Cardiac Physiome.
- We identify and classify the numerous sources of variability and uncertainty in model formulation, parameters and other inputs that arise from both natural variation in experimental data and lack of knowledge.
- The impact of uncertainty on the outputs of Cardiac Physiome models is not well understood, and this limits their utility as clinical tools.
- We argue that incorporating variability and uncertainty should be a high priority for the future of the Cardiac Physiome.
- We suggest investigating the adoption of approaches developed in other areas of science and engineering while recognising unique challenges for the Cardiac Physiome; it is likely that novel methods will be necessary that require engagement with the mathematics and statistics community.

**Abstract:**

The Cardiac Physiome effort is one of the most mature and successful applications of mathematical and computational modelling for describing and advancing the understanding of physiology. After five decades of development, physiological cardiac models are poised to realise the promise of translational research via clinical applications such as drug development and patient‐specific approaches as well as ablation, cardiac resynchronisation and contractility modulation therapies. For models to be included as a vital component of the decision process in safety‐critical applications, rigorous assessment of model credibility will be required. This White Paper describes one aspect of this process by identifying and classifying sources of variability and uncertainty in models as well as their implications for the application and development of cardiac models. We stress the need to understand and quantify the sources of variability and uncertainty in model inputs, and the impact of model structure and complexity and their consequences for predictive model outputs. We propose that the future of the Cardiac Physiome should include a probabilistic approach to quantify the relationship of variability and uncertainty of model inputs and outputs.

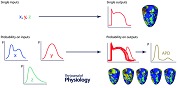

**Abbreviations**
AP: action potential
APD: action potential duration
*G*_Ks_: maximum conductance of the slow delayed rectifier current
*G*_K_: maximum conductance of outward K^+^ current
UQ: uncertainty quantification

## Introduction

The Cardiac Physiome project is an international effort to integrate different types of data across a range of time and space scales into models that encode quantitatively our understanding of cardiac physiology (Bassingthwaighte *et al*. 2009). In this approach, models are simplified representations of complex natural systems that can be used to reconstruct the behaviour of cardiac cells, tissue and the whole organ.

Models are a set of mathematical relationships, implemented in software as computational models, which produce outputs that are a function of inputs. Inputs can include model parameters, initial conditions, boundary conditions and tissue or whole organ geometry. Inputs often have physiological meaning and are typically obtained by direct measurement or indirect calibration from experimental data, or inherited from other models or model components.

Cardiac modelling has been enormously successful at yielding insight into physiological mechanisms at cell and tissue scales. For example, since the publication of the first model of cardiac cellular electrophysiology more than 50 years ago (Noble, 1962), continuous development has resulted in models with increasing biophysical detail (Fink *et al*. 2011) and has enabled important contributions to our knowledge of cardiac cellular physiology to be made. These include mechanisms of spontaneous depolarisation in sino‐atrial node cells (Noble *et al*. 1992), the role of re‐entrant spiral waves in arrhythmias (Gray *et al*. 1995), and the whole‐cell consequences of ion channel mutations (Roberts *et al*. 2012). In all these examples there was a tight integration between modelling and experimental work. Cellular level electrophysiology models are beginning to be used in safety‐critical situations such as safety pharmacology (Mirams *et al*. 2012), where they now form an integral part of a proposal to replace a human clinical pro‐arrhythmic risk trial (Sager *et al*. 2014).

Action potential models are one component of multi‐scale models of tissues and whole organs that can reconstruct the electromechanical behaviour of cardiac tissue (Trayanova, 2011), and there is the prospect of such tissue models being utilised as safety‐critical clinical tools (Relan *et al*. 2011; Sermesant *et al*. 2012; McDowell *et al*. 2013). While this is an exciting prospect, the translation from using models to test scientific hypotheses to using models to aid in clinical therapy will require the credibility of predictive model outputs to be rigorously quantified and evaluated. Establishing credibility involves an assessment of how well the model behaviour reproduces the heart function of a typical patient, as well as a consideration of how uncertainties in the model inputs and parameters influence confidence in predictive outputs for an individual patient.

In this White Paper we therefore argue that a detailed and systematic consideration of variability and uncertainty in cardiac models is an important future research direction for the Cardiac Physiome. Similar problems have been encountered in other predictive modelling fields, for example in weather forecasting, where models are also computationally intensive, multi‐scale and multi‐physics, and may be used in decisions such as whether or not to evacuate towns and cities in advance of severe weather (Bauer *et al*. 2015). A natural stage in the development and adoption of this type of computational model has been to establish a quantitative understanding of uncertainties. This has proved to be a necessary step for establishing the credibility of model predictions, especially for safety‐critical applications.

We begin by assessing the potential sources of uncertainty in cardiac models, we then highlight lessons that have been learned from other areas, enumerate some relevant mathematical tools and approaches, review recent progress in cardiac models and suggest potential areas where progress could be made. Our focus in the examples is on models of cardiac cell and tissue electrophysiology; nevertheless the principles we cover are also applicable to the rest of the Cardiac Physiome.

## Uncertainty in models of natural systems

Models of natural systems involve parameters that are either directly measured or indirectly inferred (calibrated) using experimental data. However, even the most carefully conducted experiments exhibit both *intrinsic variability* in their temporal behaviour and *extrinsic variability* between individual samples. For example, variability is reflected in the intrinsic beat‐to‐beat fluctuation of action potential duration (APD) in a single cell (Zaniboni *et al*. 2000) and extrinsic cell‐to‐cell differences in action potential duration.

Intrinsic and extrinsic variability describe fluctuations that may be due to inherent randomness, or natural differences between individuals. Variability is one cause of *uncertainty*, the confidence or precision with which a quantity can be assigned a value. Uncertainty can be either due to variability or due to lack of knowledge. Natural variation is sometimes characterised as *aleatory uncertainty*, and uncertainty arising from lack of knowledge as *epistemic uncertainty*.

Uncertainty is an important consideration not only for model calibration, where inputs such as parameter values are derived from experimental data, but also for model validation (where model outputs are evaluated against experimental data not used in the calibration stage) and for model prediction. We stress that calibration, validation and prediction are separate activities. Most models treat inputs as quantities with a fixed value, and generate outputs that are single values or a time series of single values. However, model parameters and other inputs are usually uncertain because of possible variability and the inherent limitations of experiments and calibration. This can be addressed by assigning *probability distributions* rather than fixed values to model inputs. Uncertain inputs result in uncertain model outputs, and the process for considering the impact of input uncertainties on outputs is uncertainty quantification (UQ).

**Figure 1:**
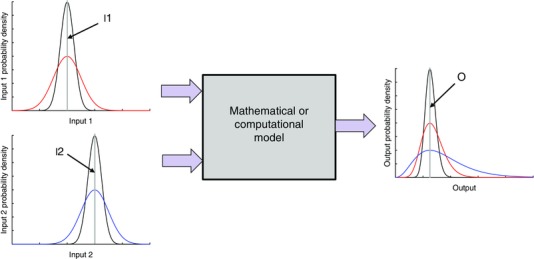
**Illustrative example showing how model inputs (I1, I2) and outputs (O) can be characterised as probability distributions rather than fixed values**

This concept is illustrated in Fig. 1, where two inputs are combined to produce an output. Grey lines indicate the conventional approach, where each input is assigned a fixed value (I1 and I2), producing a fixed value on the output (O). Uncertainty on the inputs can be expressed by assigning each input a distribution, and the black lines indicate distributions on the two inputs as well as the output distribution. In this illustrative example the input distributions are normal, but the output distributions are skewed to emphasise that it is not necessarily the case that normally distributed inputs would result in a normally distributed output. Changing the input distributions may have different effects on the model output distribution. In Fig. 1, doubling the width of the input 1 distribution has a smaller effect on the output distribution (red line) than a similar change to the width of the input 2 distribution (blue line).

UQ can be used, for example, to determine a probability distribution for an output such as APD predicted by a cardiac action potential model, based on uncertainty in selected model inputs. When the output is a time series, such as membrane voltage, then the output is a time series of probability distributions, or more precisely a stochastic process. When the output is a binary quantity, such as when whole heart models are used to address questions such as, Does ventricular tachycardia degenerate into ventricular fibrillation? UQ enables probabilities to be assigned to the discrete possibilities.

A similar and related concept is parameter sensitivity, which quantifies how sensitive model outputs are to changes in model inputs, but does not require the uncertainty in the input to be characterised. Sensitivity analysis can be used to identify model parameters and other inputs that have a dominant influence on model outputs, and so should be measured as precisely as possible (Romero *et al*. 2009; Sarkar *et al*. 2012; Pathmanathan *et al*. 2015). Conversely, sensitivity analysis can also be used to identify parameters and other inputs that do not have a strong effect on a particular output, in which case uncertainty in those inputs may be neglected in the UQ process.

**Figure 2:**
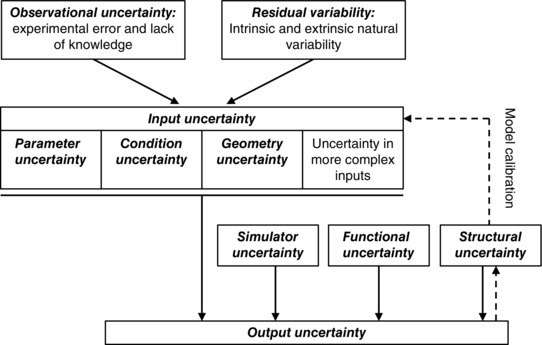
**Diagram showing how different sources of uncertainty combine to produce output uncertainty (continuous arrows), and how structural uncertainty is important for model calibration (dashed arrows)**

There is a substantial literature on uncertainty quantification (see for example Smith, 2014), and several sources of variability and uncertainty in computer models can be identified (Kennedy & O'Hagan, 2001; Vernon *et al*. 2010). These are illustrated in Fig. 2, which shows how different types of uncertainty combine to influence uncertainty in a model output.

*Observational* or *measurement uncertainty* takes into account errors in experimental measurements, and *residual variability* describes intrinsic randomness in the system as well as extrinsic variability. These types of uncertainty influence both direct parameter measurement and indirect model calibration from experiments, and contribute to *input uncertainty* in the model. Input uncertainties include *parameter uncertainty* and *condition uncertainty*, which account for uncertainties in boundary conditions and initial conditions. *Geometry uncertainty* may also arise due to observational uncertainty related to the resolution of imaging, image segmentation and mesh fitting, as well as variability in the underlying heart structure. There are often additional input uncertainties associated with more complex inputs such as the spatial distribution of various parameters including heterogeneity of cellular kinetics. The effect of the uncertainty in the model equations themselves, such as model assumptions and complexity are termed *structural uncertainty*, *model discrepancy* or *model inadequacy*. The model may be operated under conditions or with parameters that are beyond the scope of the data used to calibrate the model; this effect is termed *functional uncertainty* and sometimes *extrapolation*. When the model is implemented (usually in software) and a simulation is run, there are *simulator uncertainties* arising from numerical approximations in the implementation and in the convergence of the solver (Pathmanathan *et al*. 2012). These numerical errors and implementation uncertainties should be quantified in the calculation verification stage when the implementation of the model is verified (Pathmanathan & Gray, 2014). *Output uncertainty* is then the uncertainty in specific outputs of a deterministic computational model, given the other types of uncertainty listed above.

## Uncertainty in cardiac physiome models

These different categories of uncertainty provide a helpful framework for thinking about uncertainty propagation in cardiac models, and several important issues arise from these considerations. To further consolidate these ideas, examples of these different types of uncertainty in weather and climate models as well as Cardiac Physiome models are given in Table 1. We discuss two further examples of parameter uncertainty and condition uncertainty in detail below, and then consider other types of uncertainty.

**Table 1:** Examples of sources of uncertainty, drawing parallels between weather forecasting and cardiac modelling

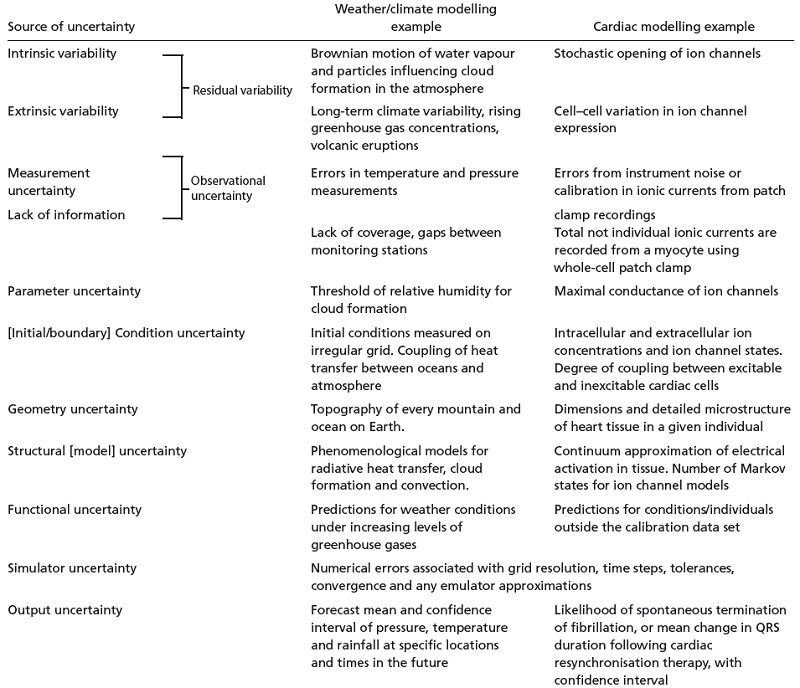

## Parameter and output uncertainty in a cardiac action potential model

**Figure 3:**
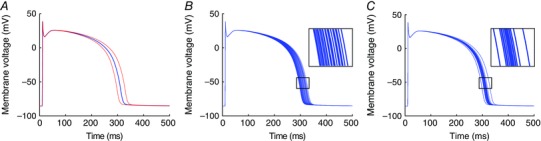
**Uncertainty propagation in an action potential model** *A*, action potentials produced by the ten Tusscher–Panfilov 2006 model when paced at a 1000 ms cycle length for 20 beats in its default configuration (blue), and with *G*_Ks_ altered by ±20% (red). *B*, a series of 20 action potentials in which the values of *G*_Ks_ are drawn from a uniform distribution with range ±20%. *C*, a series of 20 action potentials in which *G*_Ks_ is drawn from a normal distribution with a coefficient of variation (standard deviation divided by mean) of 10%.

To extend the illustrative example of input and output uncertainty shown in Fig. 1, we demonstrate the importance of parameter uncertainty with an implementation of the ten Tusscher–Panfilov 2006 model for human ventricular myocytes (ten Tusscher *et al*. 2004; ten Tusscher & Panfilov, 2006). In its default configuration for epicardial cells, the model produces an action potential at 1 Hz steady pacing with an APD of 306 ms (blue line in Fig. 3 *A*). If we were to assume that there is uncertainty of around 20% in the maximal conductance of the slow delayed rectifier current (*G*_Ks_), then we can examine the effect of this parameter uncertainty on action potentials produced by the model. In practice uncertainty in the value of *G*_Ks_ could arise from a combination of underlying residual variability and observational/measurement uncertainty in the data used to calibrate the model. A simple approach would be to simulate the action potential with *G*_Ks_ set to ±20% of its default value, and this produces action potentials with APD of 296 and 319 ms (red lines in Fig. 3 *A*). A refinement would be to run a series of simulations, each with a value of *G*_Ks_ drawn at random from this range of values. This initial refinement generates action potentials with APD uniformly distributed in the range 296–319 ms (Fig. 3 *B*). A further refinement is to select samples of *G*_Ks_ from a normal distribution, which may be a more faithful representation of parameter uncertainty and yields a different (not necessarily normal) distribution of APD (Fig. 3 *C*). For a given uncertainty in *G*_Ks_, we can therefore estimate uncertainty in APD. This is distinct from previous studies involving a population of models, where parameter space is widely and uniformly sampled and regions of parameter space leading to plausible action potentials are selected (e.g. Britton *et al*. 2013).

## Condition and output uncertainty in a cardiac tissue model

**Figure 4:**
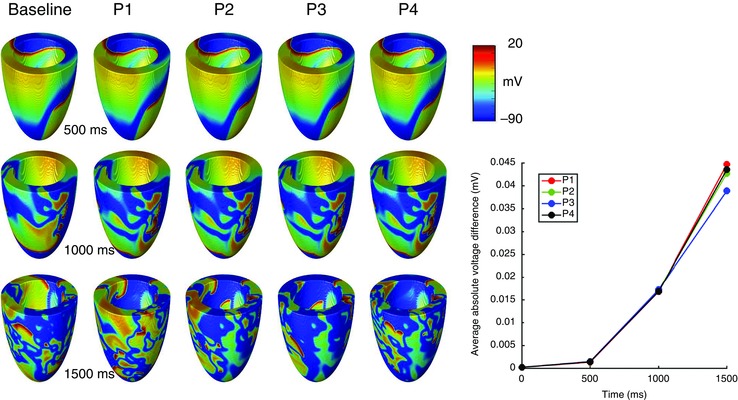
**Simulations of fibrillation in an ellipsoid geometry representing the human LV, with membrane kinetics described by a phenomenological model (Bueno‐Orovio *et al***. 2008 **) set to steep APD restitution (Clayton,** 2013 **)** Three snapshots of a baseline simulation are shown (left column), along with four further simulations (P1–P4) in which the initial conditions of a single re‐entrant wave with a transmural filament are perturbed by adding a random voltage drawn from a uniform distribution in the range ±5 mV to the initial voltage at each grid point (Qu *et al*. 2000). The graph on the right shows the nonlinear growth of the average absolute difference at each grid point between the voltage in the baseline simulation and each of the perturbations.

It is recognised that the choice of initial and boundary conditions can have an important influence on model behaviour (Fenton *et al*. 2002). Some model behaviours are highly nonlinear, and so are very sensitive to initial conditions. Figure 4 illustrates the impact of this effect on a model of re‐entry and fibrillation. The breakup of a re‐entrant wave is a nonlinear process, and perturbation of the initial conditions for the simulation has an important effect on the subsequent breakup and patterns of electrical activation.

## Other sources of uncertainty in cardiac models

Quantification of parameter uncertainty in cardiac models depends on whether the parameter can be directly measured (for example resting heart rate), or has to be indirectly inferred or calibrated using other experimental data (the vast majority of cardiac model parameters). For direct measurement, characterising and quantifying the uncertainty is a purely experimental task. For the latter, there are numerous statistical techniques for performing calibration while also accounting for uncertainty in the data to establish the uncertainty in the parameter being calibrated.

Cardiac action potential models are typically calibrated from experimental data by fitting the model to averages of experimental data without taking into account observational uncertainty or residual variability. The weakness of this approach is exposed in a recent study that shows how a simple averaging of data can result in a model that is not faithful to the data on which it is based (Pathmanathan *et al*. 2015). Given the uncertainty in data used to construct models, this raises a question about what the models actually represent.

A further complication arises because cell and tissue models are ‘modular’, and frequently make use of components (such as the description of an ion channel current) that have been developed for models of a different species or cell type. A consequence of this approach is that the provenance of model parameters is not always easy to establish (Niederer *et al*. 2009), and the consequences for parameter and structural uncertainty, as well as how these uncertainties influence the behaviour of tissue and organ scale models, are largely unknown.

Inputs that are more complex than scalar or vector parameters, for example heart geometries and anisotropy information, introduce further difficulties and contribute to geometry uncertainty (Delingette *et al*. 2012). When quantifying uncertainty in inputs that are functions of space or time (e.g. the principal fibre and sheet directions in whole‐heart models), the inputs need to be treated as random fields or random processes rather than random variables. One approach for handling this complexity is to approximate the random field using a (truncated) Karhunen–Loeve expansion (for example, see Smith, 2014), which in other settings is known as principal component analysis or proper orthogonal decomposition. This type of approach has been used to construct statistical shape models (Frangi *et al*. 2002), which address variability in heart shape and size.

For whole heart models, it is important to distinguish between the generic and patient‐specific inputs and parameters. When model inputs are patient‐specific, uncertainty results from observational error and residual variability about the properties of that individual. For generic inputs and parameters, the uncertainty is due to observational error combined with intrinsic and extrinsic variability, the latter of which may be significant and dependent on factors such as age and sex.

Cardiac action potential models have many outputs, because each state variable has a time series and so can be considered an output. However, the focus is often on membrane voltage and intracellular Ca^2+^ concentration since these outputs directly influence electrical propagation and tension generation and may be measurable. Specific features such as APD have been used to characterise the action potential (Britton *et al*. 2013; Chang *et al*. 2015), and so these can also be treated as model outputs that correspond to inputs such as model parameters and initial conditions. Larger scale models may have a scalar output such as QRS duration, a binary output such as arrhythmia or sinus rhythm, or a tensor output such as a strain field. Careful identification of the outputs that are of most importance is a key component of the UQ process.

Deeper concerns arise from considering functional and structural uncertainties. Cardiac action potential models are typically developed and parameterised from voltage‐clamp data, yet are used to reconstruct singular action potentials as well as more complex tissue behaviours such as fibrillation. Extrapolation of these models outside the parameter envelope of experimental data used in their construction is a potential source of functional uncertainty, which may be very difficult to quantify. Sources of structural uncertainty in the present generation of cardiac action potential models are the representation of Ca^2+^ storage, release and uptake. Other examples of structural uncertainty include the number of states in Markov models of ion channel behaviour, the topology of state transitions, and in tissue the difference between mono‐ or bi‐domain representations and reality. Like functional uncertainty, structural uncertainty is difficult to characterise, and is an active area of research (Kennedy & O'Hagan, 2001; Strong *et al*. 2012).

## Experience in climate modelling and weather forecasting

These concerns are not unique to cardiac models, and uncertainty quantification has a long history of application in other fields, where probabilistic approaches have been found to be essential. Early work was in engineering (Sacks *et al*. 1989; Forrester *et al*. 2008) and in flow through porous media, in particular the modelling of radioactive waste disposal and oil field reservoirs (e.g. Christie *et al*. 2006). Climate modelling has applied similar techniques (Challenor *et al*. 2010; Sexton *et al*. 2012; Williamson *et al*. 2013). Weather forecasting (Bauer *et al*. 2015) is a combination of data and model brought together in a very large data assimilation process. Because the system is believed to be chaotic, most work on uncertainty in weather forecasting has been concerned with uncertainty in the initial conditions for the forecast. Current practice in all weather forecasting centres is to run an ensemble of forecasts, each with perturbed initial conditions, with perturbations chosen to capture rapidly changing conditions. This spread gives a probabilistic prediction. In practice relatively small numbers of runs are possible due to computational costs, and the spread of predictions has often been found to be small, giving too little probability to extreme weather. To counteract this trend, more detailed representations of model uncertainty are increasingly embedded in the ensembles (Palmer, 2012), and calibration techniques can be used with observation at a series of time points to choose ensemble perturbations that produce outputs that more closely match the spread of observations (Gneiting *et al*. 2007).

## Tools for uncertainty quantification

The basis of UQ techniques is a *statistical model*, which describes a probability distribution of model output(s) as a function of uncertain model inputs (including parameters), where inputs are also probability distributions rather than fixed values. Many approaches operate within a Bayesian framework, so that the model and its outputs or predictions are conditional on inputs and assumptions.

### Monte Carlo techniques

The simplest approach to the problem of uncertainty propagation is to use Monte Carlo techniques. In this approach we sample from a statistical distribution of model inputs, simulate using the model and build up the statistical distribution of the outputs. This is the method used above in Fig. 3 to illustrate model uncertainty. The problem with Monte Carlo methods is that they are slow, requiring large numbers of runs (typically thousands) to estimate even the mean of an output distribution, let alone the full probability distribution. This is particularly true for large numbers of uncertain inputs. For larger and more complex models the problem gets worse. Thus Monte Carlo methods can soon become impractical.

This problem can be reduced with a *surrogate model* or *emulator*, a fast‐running statistical approximation of the computational model that predicts an output (or a small number of outputs) as a function of the inputs. We detail two types of emulator, which have a track record of successful use in other fields and have recently been applied to cardiac models: *polynomial chaos expansions* and *Gaussian process emulation*. Both approaches fit an emulator to a set of training data, which are model inputs and outputs obtained from a small number of model runs. The fast running emulator can then be used to make inferences about uncertainty in the model.

### Polynomial chaos expansions

The term ‘polynomial chaos’ was first coined by Wiener (1938) who studied decompositions of Brownian motion, and does not relate to non‐linearity or ‘chaos theory’. The main idea is to represent the model output as a series of polynomials in terms of the inputs, and the method was first applied to computer models by Ghanem & Spanos (1991). The polynomials are carefully chosen according to orthogonality properties and the probability distributions of the inputs. Training data are then used to determine the coefficients of the polynomial expansion. Regression methods can be used to fit the polynomial surface to training data for a general design (Berveiller *et al*. 2006). Quadrature approaches are also popular (Le Maître *et al*. 2002), but as the number of inputs increases, these methods suffer from the curse of dimensionality as the number of training data points required is large. This problem has led to the use of sparse grid methods (Xiu & Hesthaven, 2005) that can reduce the computational burden. Once the coefficients of the polynomial expansion have been found, the series of polynomials can be used to estimate model outputs for a given set of inputs.

### Gaussian process emulators

**Figure 5:**
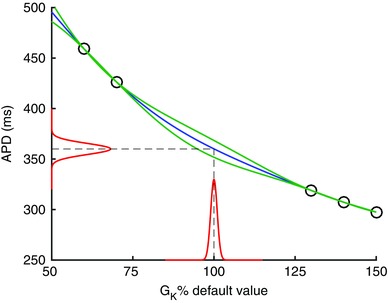
**A simple Gaussian process emulator, which relates a single output (APD) to a single input (*G*_K_) for the Luo–Rudy 1991 model (Luo & Rudy,** 1991 **)** Circles denote training data, blue line is mean of emulator, green lines are two standard deviations, red lines show distribution of output for a given input distribution. See text for further details.

An alternative to polynomial chaos also fits an output surface to the model, although the rationale is very different. This is the Gaussian process emulator, also known as a Kriging model. A good introduction to the ideas is given in O'Hagan (2006) and the methodology laid out in Challenor (2012). A Gaussian process is a continuous stochastic process, defined by a mean function and a covariance function, which produces an output that is characterised by an expectation (mean) and variance for a given set of possibly uncertain inputs (Rasmussen & Williams, 2006). As with polynomial chaos expansions, the Gaussian process is fitted to a set of training data comprising model inputs and outputs. The fitting process assumes that the output surface is smooth, without any steps or discontinuity. No other assumptions are necessary, but with a linear mean and Gaussian covariance function, as well as an assumption that both inputs and outputs are normally distributed, it is possible to calculate directly the mean and variance of outputs given the mean and variance of each uncertain input (Oakley & O'Hagan, 2002, 2004; Chang *et al*. 2015). A simple example is shown in Fig. 5, and explained in more detail below.

An important aspect of emulation is the design of the training set. We need a design that is sparse, since we assume it is expensive to run the model, but which also ‘fills’ the input space. The most common design is the Latin Hypercube (McKay *et al*. 1979), but alternatives are available such as Sobol sequences (Sobol, 1967).

Validation is also an important part of the process of building an emulator. Since emulators are built from a limited set of training data, it can be easy to build an emulator that is a poor fit to the model. To validate an emulator we need to compare the emulator output with model output for inputs that have not been used in training. There are two ways of doing this. The first is leave‐one‐out validation. Each model run in turn is removed from the training set; the emulator is then built without that point, and the missing output is then estimated using this emulator. Using the difference between the model output and the mean and variance of the emulator output, we can build up statistics on the accuracy of the emulator. An alternative is to use a separate set of validation data, and this method is explained in detail in Bastos & O'Hagan (2009).

Once we have built and validated a Gaussian process emulator there are a number of problems we can use it for. The first is predictive. The emulator can be used to predict the model output at some new set of inputs. Because the emulator includes a measure of its uncertainty we not only get an estimate of what the model would have given but also how accurate that prediction is. Beyond simple prediction the next application is sensitivity analysis. Sensitivity analysis gives the change in the output for a small change in one or more of the inputs. It is used to identify important (and not important) inputs and how these interact. Methods for sensitivity analysis using emulators are described in Oakley and O'Hagan (2004) and applied to cardiac models in Chang *et al*. (2015). The third application is UQ itself. If we are uncertain about model inputs and describe that uncertainty in terms of a probability distribution, then UQ describes how that uncertainty propagates through the model to the model outputs.

These ideas are illustrated in Fig. 5, which shows a very simple emulator where a single output is the APD of the Luo–Rudy model (Luo & Rudy, 1991), and the single input is the K^+^ channel maximum conductance *G*_K_. The emulator is fitted to five runs of the model using the approach described previously (Chang *et al*. 2015), and each run used a different value of *G*_K_. These training data are plotted as circles. The emulator then predicts a mean value of APD for any new value of *G*_K_, shown as the blue line. The predicted output is a distribution, and the green lines denote two standard deviations. At the training points, the emulator fits the data exactly, but where there are gaps between the training data the emulator output is more uncertain. An additional training point with *G*_K_ set to its default value would reduce the uncertainty of the emulator. The distributions shown in red on the *x*‐ and *y*‐axes demonstrate that if *G*_K_ is considered to be normally distributed, then we can calculate the corresponding distribution of APD.

### Model calibration

Another important application of UQ methods is model calibration or model tuning; estimating model inputs given some data on the model outputs. If we are calibrating a model, it is important to realise that models embed simplifying assumptions, and so structural uncertainty or model discrepancy becomes an important consideration. In Kennedy & O'Hagan (2001) a Gaussian process emulator is fitted to the model and at the same time a second Gaussian process is used to model the discrepancy between the model and the data. Even for simple models there are problems of identifiability with this approach because it is difficult to separate uncertainty about model parameters from model discrepancy. Brynjarsdóttir and O'Hagan (2014) show that prior information on either the model parameters or the discrepancy (or ideally both) is necessary to successfully estimate model parameters.

An alternative approach to model calibration is known as ‘history matching’ (Vernon *et al*. 2010; Williamson *et al*. 2013). Rather than trying to estimate the ‘best’ values of the parameters (or more formally their posterior distributions), history matching seeks to find those values of the model inputs that could not possibly have produced the data. This is done by using an emulator to calculate an implausibility value for all values of the inputs, which measures the scaled distance of the emulator mean from the data. The scaling depends on three terms: the variance of the data, the variance of the emulator and a model discrepancy term. The variance of the data is usually known as part of data collection, and the emulator variance is a property of the emulator. The final term is harder to define. It expresses structural uncertainty as in Kennedy & O'Hagan (2001) and must be elicited. An alternative interpretation of this term is as our tolerance to error. Any set of inputs with a value of the implausibility greater than a threshold is considered implausible and excluded from further analysis. This exclusion is done in a sequential way, and at each ‘wave’ more runs of the model are produced in the ‘not implausible region’. Building new emulators at each wave reduces the emulator uncertainty and refines the implausibility measure. Eventually the not implausible region no longer contracts and either more accurate data or a greater tolerance to error is required to reduce the region further.

## Recent developments

Applications of these tools and approaches to cardiac models are in their infancy, but there has been some important initial progress.

Many studies have been published on different methods to fit a point parameter estimate in cardiac models, all of which involve some kind of optimisation procedure resulting in a single optimal parameter set. Of particular note amongst these studies are those that suggest new experiments to assist in the parameter fitting process, i.e. reduce epistemic/observational uncertainty (see e.g. Dokos & Lovell, 2004; Kaur *et al*. 2014; Groenendaal *et al*. 2015). A formal methodology for experimental design based on various ‘optimality criteria’ has a long history in the statistics and control theory literature (Goodwin & Payne, 1977). Fink & Noble (2009) applied one such method to design novel voltage clamp protocols that are optimised to identify parameters in ion current models, with the added benefit of an experiment with a shorter duration than traditional methods; and other studies have developed ideas based on analysis of the Hodgkin–Huxley model (Raba *et al*. 2013). These formal methods have also been applied in systems biology and extended to address model selection (see e.g. Liepe *et al*. 2013; Silk *et al*. 2014), and so there are grounds to believe the application of optimal experimental design to cardiac modelling will be very fruitful.

Within cardiac modelling, the sensitivity of outputs to variations in parameter sets, or the model formulation, has also been examined (see e.g. Romero *et al*. 2009; Huberts *et al*. 2013 *a*,*b*; Bear *et al*. 2015). Other approaches have taken a range of possible parameter values calibrated against experimental recordings to capture experimental variability (e.g. Sarkar & Sobie, 2011; Sarkar *et al*. 2012; Britton *et al*. 2013). Below we will highlight a selection of studies that take a probabilistic approach to variability, or attach probability distributions to parameter values, which will be necessary to enable rigorous uncertainty quantification.

The intrinsic variability of ion currents, due to the stochastic opening and closing of ion channels, has been studied in some detail in papers such as Geneser *et al*. (2007), Pueyo *et al*. (2011) and Heijman *et al*. (2013). These studies examine the consequences of the intrinsic variability on behaviour such as macroscopic currents, beat‐to‐beat variability of APs, and the emergence of pro‐arrhythmic behaviour. More of this type of work is required to examine the situations under which the consequences of intrinsic variability need to be taken into consideration, and to generate computationally simple ways to capture the effects of intrinsic variability without having to simulate the activity of every individual ion channel.

Ion channel state transition parameters were given probability distributions in a study by Siekmann *et al*. (2011), and the authors also showed how Bayesian inference could assist in studying epistemic uncertainty (Siekmann *et al*. 2012). The ion current densities (‘maximum conductances’ for ion channels) are perhaps the most important determinants of cellular‐scale electrical properties and variability between cell types and species. Sarkar & Sobie (2010) explored the use of Bayesian inference to attach a probability distribution to current densities, given different datasets, and explored how variation in densities might explain extrinsic variability between patients.

Studies have been performed where inputs to action potential simulations were given probability distributions, and simple Monte Carlo uncertainty propagation was performed to quantify uncertainty on model outputs (Elkins *et al*. 2013). Surrogate models have been applied to make this process fast and cheap to calculate (simple lookup table interpolation was used in Mirams *et al*. (2014), and a Gaussian Process emulator in Chang *et al*. (2015)). Pathmanathan *et al*. (2015) performed perhaps the first multi‐scale uncertainty quantification study in cardiac electrophysiology: identifying the variability in fast sodium inactivation curves between individuals and the uncertainty in the population average, and propagating this through to both AP and tissue simulations, to examine the influence of variability at the sodium channel on emergent behaviour at different scales.

Uncertainty calculations can also be performed for spatial problems (see e.g. Xiu & Sherwin, 2007; Konukoglu *et al*. 2011; Wallman *et al*. 2014), and special methods to visualize the results of these have also been developed (Burton *et al*. 2013). Cardiac geometry atlases have included statistical measures of variability for some time (Frangi *et al*. 2002), and so many of the tools are already in place to examine the consequences of variable tissue geometry and properties on tissue‐level simulation results.

## Opportunities, challenges and future directions

In this White Paper, we have argued the importance of uncertainty and variability in the Cardiac Physiome, and the need for techniques and approaches that can quantify confidence in model predictions. We consider this to be a critical next step, especially for models that could be deployed in safety critical applications.

Two opportunities are immediately apparent. The first is to benefit from links to other communities with experience of working on related problems. In particular, the statistics community have developed tools and approaches for handling uncertainty in multi‐scale and computationally expensive models, and there is enthusiasm for engagement with a new and challenging set of problems. The second opportunity is for Cardiac Physiome models to become far more robust because they take account of uncertainty, enabling not only improved hypothesis testing for basic science, but also greater suitability for clinical applications.

Cardiac models are highly detailed, and adapting existing modelling paradigms and software to take account of uncertainty is a significant challenge. Throughout this White Paper we have highlighted specific examples in cardiac models where uncertainty is important, although we have not attempted a full systematic analysis. Nevertheless, it is clear that there are very many sources of uncertainty and variability, and another major challenge is to enumerate these carefully. We consider it highly likely that application of existing tools and techniques for uncertainty quantification to cardiac models will unearth new mathematical and statistical questions, and so serious engagement with mathematicians and statisticians will be essential in this process.

Consideration of uncertainty in Cardiac Physiome models is therefore an important future research direction. There is much to accomplish, and we identify the following as important research questions:

- *How reliable are the present generation of action potential models?* An answer to this question will involve systematic analysis of input uncertainties, including an assessment of how model parameters were fitted to data, as well as the effect of assumptions and simplifications in model components, in particular Markov state models of ion channels, and components of the Ca^2+^ handling system.
- *Can we compare action potential models in a rigorous way?* Many different action potential models have been developed, often for the same species and cell type, yet can show different behaviours (Cherry & Fenton, 2007; Cooper *et al*. 2016). The UQ approaches we have described have the potential to offer a rigorous and quantitative framework in which the behaviour action potential models can be compared with each other, as well as with experimental data.
- *How do uncertainties at the cell scale contribute to uncertainties in tissue scale models?* Propagation of uncertainties is a critical question for multi‐scale cardiac models because there are many situations where tissue scale responses might be sensitive to cell scale behaviour (APD restitution, Ca^2+^ handling, tension generation) combined with tissue scale properties (tissue conductivities, passive mechanical properties, tissue microstructure, distribution of cell types). Examples would include the onset of alternans and the stability of re‐entry for models of electrophysiology, and deformation sequence and myocardial work in models of mechanics.
- *What criteria should be used to choose a cell, tissue and geometrical model for a particular context of use?* At present, the choice of model is often pragmatic, based on personal preference and the imaging modalities and codes that are available. However, reliable estimates of model output uncertainties potentially enable a rational scheme for model selection based on a trade‐off of output uncertainty against computational cost. Context of use will establish the output uncertainties that are acceptable, with more stringent requirements for safety‐critical applications such as guidance for catheter ablation.
- *How should uncertainties in Cardiac Physiome models be visualised and communicated to users such as clinicians?* The clinical environment can be characterised as data rich and information poor. Clinicians are often provided with overwhelming data, and for Cardiac Physiome models to make an impact as clinical tools, it will be important to communicate uncertainty and model credibility clearly.

As these and other research questions are addressed, we expect that Cardiac Physiome models will not only continue to make important contributions to basic science physiology, but also be deployed in clinical tools and applications for the benefit of human health.

## Additional information

### Competing interests

None declared.

### Author contributions

G.R.M., P.P. and R.H.C. conceived and designed the paper; R.A.G. and P.C. contributed material and ideas; all authors analysed and refined both text and intellectual content. All authors have approved the final version of the manuscript and agree to be accountable for all aspects of the work. All persons designated as authors qualify for authorship, and all those who qualify for authorship are listed.

### Funding

G.R.M. gratefully acknowledges support from a Sir Henry Dale Fellowship jointly funded by the Wellcome Trust and the Royal Society (Grant Number 101222/Z/13/Z). R.H.C. gratefully acknowledges funding from the UK Engineering and Physical Sciences Research Council (Grant Numbers EP/K037145/1 and EP/L001101/1). The mention of commercial products, their sources, or their use in connection with material reported here is not to be construed as either an actual or implied endorsement of such products by the Department of Health and Human Services.

### Resources

The Managing Uncertainty in Complex Models web pages (www.mucm.ac.uk) provide a good background to the tools and approaches discussed in this White Paper, as well as others. The MUCM Toolkit (http://mucm.aston.ac.uk) is an on‐line resource that gives the mathematical underpinning to Gaussian process emulators and hints on how to do the computation. Software resources that implement some of these techniques include https://dakota.sandia.gov/, http://dice.emse.fr/ (in French), http://www.uqlab.com and https://github.com/SheffieldML/GPy.

## References

[tjp7214-bib-0001] Bassingthwaighte J , Hunter P & Noble D (2009). The cardiac physiome: perspectives for the future. Exp Physiol 94, 597–605. 19098089 10.1113/expphysiol.2008.044099 PMC2854146

[tjp7214-bib-0002] Bastos LS & O'Hagan A (2009). Diagnostics for Gaussian process emulators. Technometrics 51, 425–438.

[tjp7214-bib-0003] Bauer P , Thorpe A & Brunet G (2015). The quiet revolution of numerical weather prediction. Nature 525, 47–55. 26333465 10.1038/nature14956

[tjp7214-bib-0004] Bear LR , Cheng LK , LeGrice IJ , Sands GB , Lever NA , Paterson DJ & Smaill BH (2015). Forward problem of electrocardiography: is it solved? Circ Arrhythm Electrophysiol 8, 677–684. 25834182 10.1161/CIRCEP.114.001573

[tjp7214-bib-0005] Berveiller M , Sudret B & Lemaire M (2006). Stochastic finite element: a non intrusive approach by regression. Eur J Comput Mech 15, 81–92.

[tjp7214-bib-0006] Britton OJ , Bueno‐Orovio A , Van Ammel K , Lu HR , Towart R , Gallacher DJ & Rodriguez B (2013). Experimentally calibrated population of models predicts and explains intersubject variability in cardiac cellular electrophysiology. Proc Natl Acad Sci USA 110, E2098–E2105. 23690584 10.1073/pnas.1304382110 PMC3677477

[tjp7214-bib-0007] Brynjarsdóttir J & OʼHagan A (2014). Learning about physical parameters: the importance of model discrepancy. Inverse Probl 30, 114007.

[tjp7214-bib-0008] Bueno‐Orovio A , Cherry EM & Fenton FH (2008). Minimal model for human ventricular action potentials in tissue. J Theor Biol 253, 544–560. 18495166 10.1016/j.jtbi.2008.03.029

[tjp7214-bib-0009] Burton BM , Erem B , Potter K , Rosen P , Johnson CR , Brooks DH & Macleod RS (2013). Uncertainty visualization in forward and inverse cardiac models. Comput Cardiol 40, 57–60. PMC4221850 25383390

[tjp7214-bib-0010] Challenor P (2012). Using emulators to estimate uncertainty in complex models In Uncertainty Quantification in Scientific Computing, ed. Dienstfrey AM & Boisvert RF, pp. 151–164. Springer‐Verlag, Berlin.

[tjp7214-bib-0011] Challenor P , McNeall D & Gattiker J (2010). Assessing the probability of rare climate events In The Oxford Handbook of Applied Bayesian Analysis, ed. O'Hagan A & West M, pp. 403–430. Oxford University Press, Oxford.

[tjp7214-bib-0012] Chang ETY , Strong M & Clayton RH (2015). Bayesian sensitivity analysis of a cardiac cell model using a Gaussian process emulator. PLoS One 10, e0130252. 26114610 10.1371/journal.pone.0130252 PMC4482712

[tjp7214-bib-0013] Cherry EM & Fenton FH (2007). A tale of two dogs. Analyzing two models of canine ventricular electrophysiology. Am J Physiol Heart Circ Physiol 292, H43–H55. 10.1152/ajpheart.00955.2006 16997886

[tjp7214-bib-0014] Christie M , Demyanov V & Erbas D (2006). Uncertainty quantification for porous media flows. J Comput Physics 217, 143–158.

[tjp7214-bib-0015] Clayton RH (2013). Models of ventricular arrhythmia mechanisms. Conf Proc IEEE Eng Med Biol Soc 2013, 1526–1529. 24109990 10.1109/EMBC.2013.6609803

[tjp7214-bib-0016] Cooper J , Scharm M & Mirams GR (2016). The Cardiac Electrophysiology Web Lab. Biophys J 110, 292–300. 26789753 10.1016/j.bpj.2015.12.012 PMC4724653

[tjp7214-bib-0017] Delingette H , Billet F , Wong K , Sermesant M , Rhode K , Ginks M , Rinaldi C , Razavi R & Ayache N (2012). Personalization of cardiac motion and contractility from images using variational data assimilation. IEEE Trans Biomed Eng 59, 1–4. 10.1109/TBME.2011.2160347 21712158

[tjp7214-bib-0018] Dokos S & Lovell NH (2004). Parameter estimation in cardiac ionic models. Prog Biophys Mol Biol 85, 407–431. 15142755 10.1016/j.pbiomolbio.2004.02.002

[tjp7214-bib-0019] Elkins RC , Davies MR , Brough SJ , Gavaghan DJ , Cui Y , Abi‐Gerges N , Mirams GR (2013). Variability in high‐throughput ion‐channel screening data and consequences for cardiac safety assessment. J Pharm Toxicol Methods 68, 112–122. 10.1016/j.vascn.2013.04.007 PMC4135079 23651875

[tjp7214-bib-0020] Fenton FH , Cherry EM , Hastings HM & Evans SJ (2002). Multiple mechanisms of spiral wave breakup in a model of cardiac electrical activity. Chaos 12, 852–892. 12779613 10.1063/1.1504242

[tjp7214-bib-0021] Fink M , Niederer SA , Cherry EM , Fenton FH , Koivumaki JT , Seemann G , Thul R , Zhang H , Sachse FB , Crampin EJ & Smith NP (2011). Cardiac cell modelling: Observations from the heart of the cardiac physiome project. Prog Biophys Mol Biol 104, 2–21. 20303361 10.1016/j.pbiomolbio.2010.03.002

[tjp7214-bib-0022] Fink M & Noble D (2009). Markov models for ion channels: versatility versus identifiability and speed. Philos Trans A Math Phys Eng Sci 367, 2161–2179. 19414451 10.1098/rsta.2008.0301

[tjp7214-bib-0023] Forrester A , Sobester A & Keane A (2008). Engineering Design via Surrogate Modelling: A Practical Guide, p. 228 Wiley, Chichester, UK.

[tjp7214-bib-0024] Frangi AF , Rueckert D , Schnabel JA & Niessen WJ (2002). Automatic construction of multiple‐object three‐dimensional statistical shape models: application to cardiac modeling. IEEE Trans Med Imaging 21, 1151–1166. 12564883 10.1109/TMI.2002.804426

[tjp7214-bib-0025] Geneser SE , Kirby RM , Xiu D & Sachse FB (2007). Stochastic Markovian modeling of electrophysiology of ion channels: Reconstruction of standard deviations in macroscopic currents. J Theor Biol 245, 627–637. 17204291 10.1016/j.jtbi.2006.10.016

[tjp7214-bib-0026] Ghanem RG & Spanos PD (1991). Stochastic Finite Elements: A Spectral Approach. Springer‐Verlag, Berlin.

[tjp7214-bib-0027] Goodwin GC & Payne RL (1977). Dynamic System Identification: Experiment Design and Data Analysis. Academic Press, New York.

[tjp7214-bib-0028] Gneiting T , Balabdaoui F & Raftery AE (2007). Probabilistic forecasts, calibration and sharpness. J Roy Statist Soc B 69, 243–68.

[tjp7214-bib-0029] Gray RA , Jalife J , Panfilov AV , Baxter WT , Cabo C , Davidenko JM & Pertsov AM (1995). Nonstationary vortexlike reentrant activity as a mechanism of polymorphic ventricular tachycardia in the isolated rabbit heart. Circulation 91, 2454–2469. 7729033 10.1161/01.cir.91.9.2454

[tjp7214-bib-0030] Groenendaal W , Ortega FA , Kherlopian AR , Zygmunt AC , Krogh‐Madsen T & Christini DJ (2015). Cell‐specific cardiac electrophysiology models. PLoS Comput Biol 11, e1004242. 25928268 10.1371/journal.pcbi.1004242 PMC4415772

[tjp7214-bib-0031] Heijman J , Zaza A , Johnson DM , Rudy Y , Peeters RLM , Volders PGA & Westra RL (2013). Determinants of beat‐to‐beat variability of repolarization duration in the canine ventricular myocyte: a computational analysis. PLoS Comput Biol 9, e1003202. 23990775 10.1371/journal.pcbi.1003202 PMC3749940

[tjp7214-bib-0032] Huberts W , de Jonge C , van der Linden WPM , Inda MA , Tordoir JHM , van de Vosse FN & Bosboom EMH (2013 *a*). A sensitivity analysis of a personalized pulse wave propagation model for arteriovenous fistula surgery. Part A: Identification of most influential model parameters. Med Eng Phys 35, 810–826. 22964062 10.1016/j.medengphy.2012.08.013

[tjp7214-bib-0033] Huberts W , de Jonge C , van der Linden WPM , Inda MA , Passera K , Tordoir JHM , van de Vosse FN & Bosboom EMH (2013 *b*). A sensitivity analysis of a personalized pulse wave propagation model for arteriovenous fistula surgery. Part B: Identification of possible generic model parameters. Med Eng Phys 35, 827–837. 22964064 10.1016/j.medengphy.2012.08.012

[tjp7214-bib-0034] Kaur J , Nygren A & Vigmond EJ (2014). Fitting membrane resistance along with action potential shape in cardiac myocytes improves convergence: Application of a multi‐objective parallel genetic algorithm. PLoS One 9, e107984. 25250956 10.1371/journal.pone.0107984 PMC4176019

[tjp7214-bib-0035] Kennedy MC & O'Hagan A (2001). Bayesian calibration of computer models. J R Stat Soc Ser B Stat Methodol 63, 425–464.

[tjp7214-bib-0036] Konukoglu E , Relan J , Cilingir U , Menze BH , Chinchapatnam P , Jadidi A , Cochet H , Hocini M , Delingette H , Jaïs P , Haïssaguerre M , Ayache N & Sermesant M (2011). Efficient probabilistic model personalization integrating uncertainty on data and parameters: Application to eikonal‐diffusion models in cardiac electrophysiology. Prog Biophys Mol Biol 107, 134–146. 21763715 10.1016/j.pbiomolbio.2011.07.002

[tjp7214-bib-0037] Le Maître OP , Reagan MT , Najm HN , Ghanem RG & Knio OM (2002). A stochastic projection method for fluid flow: 2. Random process. J Comput Physics 181, 9–44.

[tjp7214-bib-0038] Liepe J , Filippi S , Komorowski M & Stumpf MPH (2013). Maximizing the information content of experiments in systems biology. PLoS Comput Biol 9, e1002888. 23382663 10.1371/journal.pcbi.1002888 PMC3561087

[tjp7214-bib-0039] Luo CH & Rudy Y (1991). A model of the ventricular cardiac action potential. Depolarization, repolarization, and their interaction. Circ Res 68, 1501–1526. 1709839 10.1161/01.res.68.6.1501

[tjp7214-bib-0040] McDowell KS , Vadakkumpadan F , Blake R , Blauer J , Plank G , Macleod RS & Trayanova NA (2013). Mechanistic inquiry into the role of tissue remodeling in fibrotic lesions in human atrial fibrillation. Biophys J 104, 2764–2773. 23790385 10.1016/j.bpj.2013.05.025 PMC3686346

[tjp7214-bib-0041] McKay MD , Beckman RJ & Conover WJ (1979). A comparison of three methods for selecting values of input variables in the analysis of output from a computer code. Technometrics 21, 239–245.

[tjp7214-bib-0042] Mirams GR , Davies MR , Brough SJ , Bridgland‐Taylor MH , Cui Y , Gavaghan DJ & Abi‐Gerges N (2014). Prediction of thorough QT study results using action potential simulations based on ion channel screens. J Pharm Toxicol Methods 70, 246–254. 10.1016/j.vascn.2014.07.002 PMC4266452 25087753

[tjp7214-bib-0043] Mirams GR , Davies MR , Cui Y , Kohl P & Noble D (2012). Application of cardiac electrophysiology simulations to pro‐arrhythmic safety testing. Br J Pharmacol 167, 932–945. 22568589 10.1111/j.1476-5381.2012.02020.x PMC3492977

[tjp7214-bib-0044] Niederer SA , Fink M , Noble D & Smith NP (2009). A meta‐analysis of cardiac electrophysiology computational models. Exp Physiol 94, 486–495. 19139063 10.1113/expphysiol.2008.044610

[tjp7214-bib-0045] Noble D (1962). A modification of the Hodgkin–Huxley equations applicable to Purkinje fibre action and pacemaker potentials. J Physiol 160, 317–352. 14480151 10.1113/jphysiol.1962.sp006849 PMC1359535

[tjp7214-bib-0046] Noble D , Denyer JC , Brown HF & DiFrancesco D (1992). Reciprocal role of the inward currents ib, Na and i(f) in controlling and stabilizing pacemaker frequency of rabbit sino‐atrial node cells. Proc Biol Sci 250, 199–207. 1283636 10.1098/rspb.1992.0150

[tjp7214-bib-0047] Oakley JE & O'Hagan A (2002). Bayesian inference for the uncertainty distribution of computer model outputs. Biometrika 89, 769–84.

[tjp7214-bib-0048] Oakley JE & O'Hagan A (2004). Probabilistic sensitivity analysis of complex models: a Bayesian approach. J R Stat Soc Series B Stat Methodol 66, 751–69.

[tjp7214-bib-0049] O'Hagan A (2006). Bayesian analysis of computer code outputs: A tutorial. Reliab Eng Syst Safe 91, 1290–1300.

[tjp7214-bib-0050] Palmer TN (2012). Towards the probabilistic Earth‐system simulator: a vision for the future of climate and weather prediction. Q J Roy Meteorol Soc 138, 841–861.

[tjp7214-bib-0051] Pathmanathan P , Bernebeu MO , Niederer SA , Gavaghan DJ & Kay D (2012). Computational modelling of cardiac electrophysiology: explanation of the variability of results from different numerical solvers. Int J Numer Method Biomed Eng 28, 890–903. 25099569 10.1002/cnm.2467

[tjp7214-bib-0052] Pathmanathan P & Gray R (2014). Verification of computational models of cardiac electrophysiology. Int J Numer Method Biomed Eng 30, 525–544. 24259465 10.1002/cnm.2615

[tjp7214-bib-0053] Pathmanathan P , Shotwell MS , Gavaghan DJ , Cordeiro JM & Gray RA (2015). Uncertainty quantification of fast sodium current steady‐state inactivation for multi‐scale models of cardiac electrophysiology. Prog Biophys Mol Biol 117, 1–15. 25661325 10.1016/j.pbiomolbio.2015.01.008 PMC4472478

[tjp7214-bib-0054] Pueyo E , Corrias A , Virág L , Jost N , Szél T , Varró A , Szentandrássy N , Nánási PP , Burrage K & Rodríguez B (2011). A multiscale investigation of repolarization variability and its role in cardiac arrhythmogenesis. Biophys J 101, 2892–2902. 22208187 10.1016/j.bpj.2011.09.060 PMC3244062

[tjp7214-bib-0055] Qu Z , Kil J , Xie F , Garfinkel A & Weiss J (2000). Scroll wave dynamics in a three‐dimensional cardiac tissue model: roles of restitution, thickness, and fiber rotation. Biophys J 78, 2761–2775. 10827961 10.1016/S0006-3495(00)76821-4 PMC1300866

[tjp7214-bib-0056] Raba AE , Cordeiro JM , Antzelevitch C & Beaumont J (2013). Extending the conditions of application of an inversion of the Hodgkin‐Huxley gating model. Bull Math Biol 75, 752–573. 23595789 10.1007/s11538-013-9832-7 PMC3855235

[tjp7214-bib-0057] Rasmussen CE & Williams CKI (2006). Gaussian Processes for Machine Learning. MIT Press, Cambridge, MA.

[tjp7214-bib-0058] Relan J , Chinchapatnam P & Sermesant M (2011). Coupled personalization of cardiac electrophysiology models for prediction of ischaemic ventricular tachycardia. Interface Focus 1, 396–407. 22670209 10.1098/rsfs.2010.0041 PMC3262447

[tjp7214-bib-0059] Roberts BN , Yang P‐C , Behrens SB , Moreno JD & Clancy CE (2012). Computational approaches to understand cardiac electrophysiology and arrhythmias. Am J Physiol Heart Circ Physiol 303, H766–H783. 22886409 10.1152/ajpheart.01081.2011 PMC3774200

[tjp7214-bib-0060] Romero L , Pueyo E , Fink M , Rodriguez B & Rodríguez B (2009). Impact of ionic current variability on human ventricular cellular electrophysiology. Am J Physiol Heart Circ Physiol 297, H1436–H1445. 19648254 10.1152/ajpheart.00263.2009

[tjp7214-bib-0061] Sacks J , Welch WJ , Mitchell TJ & Wynn HP (1989). Design and analysis of computer experiments. Stat Sci 4, 409–435.

[tjp7214-bib-0062] Sager PT , Gintant G , Turner JR , Pettit S & Stockbridge N (2014). Rechanneling the cardiac proarrhythmia safety paradigm: A meeting report from the Cardiac Safety Research Consortium. Am Heart J 167, 292–300. 24576511 10.1016/j.ahj.2013.11.004

[tjp7214-bib-0063] Sarkar AX , Christini DJ & Sobie EA (2012). Exploiting mathematical models to illuminate electrophysiological variability between individuals. J Physiol 590, 2555–2567. 22495591 10.1113/jphysiol.2011.223313 PMC3424714

[tjp7214-bib-0064] Sarkar AX & Sobie EA (2010). Regression analysis for constraining free parameters in electrophysiological models of cardiac cells. PLoS Comput Biol 6, e1000914. 20824123 10.1371/journal.pcbi.1000914 PMC2932676

[tjp7214-bib-0065] Sarkar AX & Sobie EA (2011). Quantification of repolarization reserve to understand interpatient variability in the response to proarrhythmic drugs: a computational analysis. Heart Rhythm 8, 1749–1755. 21699863 10.1016/j.hrthm.2011.05.023 PMC3202650

[tjp7214-bib-0066] Sermesant M , Chabiniok R , Chinchapatnam P , Mansi T , Billet F , Moireau P , Peyrat JM , Wong K , Relan J , Rhode K , Ginks M , Lambiase P , Delingette H , Sorine M , Rinaldi CA , Chapelle D , Razavi R & Ayache N (2012). Patient‐specific electromechanical models of the heart for the prediction of pacing acute effects in CRT: A preliminary clinical validation. Med Image Anal 16, 201–215. 21920797 10.1016/j.media.2011.07.003

[tjp7214-bib-0067] Sexton DMH , Murphy JM , Collins M & Webb MJ (2012). Multivariate probabilistic projections using imperfect climate models part I: Outline of methodology. Clim Dynam 38, 2513–2542.

[tjp7214-bib-0068] Siekmann I , Sneyd J & Crampin EJ (2012). MCMC can detect nonidentifiable models. Biophys J 103, 2275–2286. 23283226 10.1016/j.bpj.2012.10.024 PMC3514526

[tjp7214-bib-0069] Siekmann I , Wagner LE , Yule D , Fox C , Bryant D , Crampin EJ & Sneyd J (2011). MCMC estimation of Markov models for ion channels. Biophys J 100, 1919–1929. 21504728 10.1016/j.bpj.2011.02.059 PMC3077709

[tjp7214-bib-0070] Silk D , Kirk PDW , Barnes CP , Toni T & Stumpf MPH (2014). Model selection in systems biology depends on experimental design. PLoS Comput Biol 10, e1003650. 24922483 10.1371/journal.pcbi.1003650 PMC4055659

[tjp7214-bib-0071] Smith RC (2014). Uncertainty Quantification. Theory, Implementation, and Applications. SIAM Computational Science and Engineering, Philadelphia.

[tjp7214-bib-0072] Sobol IM (1967). On the distribution of points in a cube and the approximate evaluation of integrals. USSR Comp Math Math+ 7, 86–112.

[tjp7214-bib-0073] Strong M , Oakley JE & Chilcott J (2012). Managing structural uncertainty in health economic decision models: a discrepancy approach. J R Stat Soc Ser C Appl Stat 61, 25–45.

[tjp7214-bib-0074] Ten Tusscher KHWJ , Noble D , Noble PJ & Panfilov AV (2004). A model for human ventricular tissue. Am J Physiol Heart Circ Physiol 286, H1573–H1589. 14656705 10.1152/ajpheart.00794.2003

[tjp7214-bib-0075] Ten Tusscher KHWJ & Panfilov AV (2006). Alternans and spiral breakup in a human ventricular tissue model. Am J Physiol Heart Circ Physiol 291, 1088–1100. 10.1152/ajpheart.00109.2006 16565318

[tjp7214-bib-0076] Trayanova NA (2011). Whole‐heart modeling: applications to cardiac electrophysiology and electromechanics. Circ Res 108, 113–128. 21212393 10.1161/CIRCRESAHA.110.223610 PMC3031963

[tjp7214-bib-0077] Vernon I , Goldstein M & Bower RG (2010). Galaxy formation: a Bayesian uncertainty analysis. Bayesian Anal 5, 619–669.

[tjp7214-bib-0083] Wallman M , Smith NP & Rodriguez B (2014). Computational methods to reduce uncertainty in the estimation of cardiac conduction properties from electroanatomical recordings. Med Image Anal 18, 228–240. 24247034 10.1016/j.media.2013.10.006

[tjp7214-bib-0078] Wiener N (1938). The homogeneous chaos. Am J Math 60, 897–936.

[tjp7214-bib-0079] Williamson D , Goldstein M , Allison L , Blaker A , Challenor P , Jackson L & Yamazaki K (2013). History matching for exploring and reducing climate model parameter space using observations and a large perturbed physics ensemble. Climate Dynamics 41, 1703–1729.

[tjp7214-bib-0080] Xiu D & Hesthaven JS (2005). High‐order collocation methods for differential equations with random inputs. SIAM J Sci Comput 27, 1118–1139.

[tjp7214-bib-0081] Xiu D & Sherwin SJ (2007). Parametric uncertainty analysis of pulse wave propagation in a model of a human arterial network. J Comput Physics 226, 1385–1407.

[tjp7214-bib-0082] Zaniboni M , Pollard AE , Yang L & Spitzer KW (2000). Beat‐to‐beat repolarization variability in ventricular myocytes and its suppression by electrical coupling. Am J Physiol Heart Circ Physiol 278, H677–H687. 10710334 10.1152/ajpheart.2000.278.3.H677

